# Eye Movements in Frogs and Salamanders—Testing the Palatal Buccal Pump Hypothesis

**DOI:** 10.1093/iob/obz011

**Published:** 2019-06-07

**Authors:** F Witzmann, E L Brainerd, N Konow

**Affiliations:** 1 Museum für Naturkunde, Leibniz Institute for Evolution and Biodiversity Science, Invalidenstrasse 43, 10115 Berlin, Germany; 2 Department of Ecology and Evolutionary Biology, Brown University, Providence, RI 02912 USA; 3 Department of Biological Sciences, UMass Lowell, Lowell MA 01854 USA

## Abstract

In frogs and salamanders, movements of the eyeballs in association with an open palate have often been proposed to play a functional role in lung breathing. In this “palatal buccal pump,” the eyeballs are elevated during the lowering of the buccal floor to suck air in through the nares, and the eyeballs are lowered during elevation of the buccal floor to help press air into the lungs. Here, we used X-Ray Reconstruction of Moving Morphology to investigate eye movements during lung breathing and feeding in bullfrogs and axolotls. Our data do not show eye movements that would be in accordance with the palatal buccal pump. On the contrary, there is a small passive elevation of the eyeballs when the buccal floor is raised. Inward drawing of the eyeballs occurs only during body motion and for prey transport in bullfrogs, but this was not observed in axolotls. Each eye movement in bullfrogs has a vertical, a mediolateral, and an anteroposterior component. Considering the surprisingly weak posterior motion component of the eyeballs, their main role in prey transport might be fixing the prey by pressing it against the buccal floor. The retraction of the buccal floor would then contribute to the posterior push of the prey. Because our study provides no evidence for a palatal buccal pump in frogs and salamanders, there is also no experimental support for the idea of a palatal buccal pump in extinct temnospondyl amphibians, in contrast to earlier suggestions.

## Synopsis

Augenbewegungen bei Fröschen und Salamandern-Prüfung der “palatalen Bukkalpumpen”-Hypothese Bei Fröschen und Salamandern wurde oft vorgeschlagen, dass Bewegungen der Augäpfel in Verbindung mit einem offenen Gaumen eine funktionale Rolle bei der Lungenatmung spielen. Bei dieser “palatalen Bukkalpumpe” werden die Augäpfel während des Absenkens des Mundbodens angehoben, um Luft durch die Nasenlöcher anzusaugen, und die Augäpfel werden während des Anhebens des Mundbodens abgesenkt, um Luft in die Lungen zu drücken. Hier verwendeten wir Röntgenrekonstruktionen der Bewegungsmorphologie, um Augenbewegungen während der Lungenatmung und bei der Ernährung von Ochsenfröschen und Axolotl zu untersuchen. Unsere Daten zeigen keine Augenbewegungen, die der palatalen Bukkalpumpe entsprechen würden. Im Gegenteil, es gibt eine kleine passive Anhebung der Augäpfel, wenn der Mundboden angehoben wird. Ein nach innen Ziehen der Augäpfel tritt nur während der Körperbewegung und für den Beutetransport in Ochsenfröschen auf, bei Axolotl wurde dies jedoch nicht beobachtet. Jede Augenbewegung in Ochsenfröschen hat eine vertikale, eine mediolaterale und eine anteroposteriore Komponente. Angesichts der überraschend schwachen posterioren Bewegungskomponente der Augäpfel könnte ihre Hauptaufgabe beim Beutetransport die Fixierung der Beute sein, indem sie gegen den bukkalen Mundboden Boden gedrückt wird. Das Zurückziehen des Mundbodens würde dann zum nach hinten gerichteten Schub der Beute beitragen. Da unsere Studie keine Belege für eine palatale Bukkalpumpe in Fröschen und Salamandern liefert, gibt es im Gegensatz zu früheren Vorschlägen auch keine experimentelle Unterstützung für die Idee einer palatalen Bukkalpumpe in ausgestorbenen temnospondylen Amphibien. translated to German by F. Klimm (frederike.klimm@neptun.uni-freiburg.de)

حركات العين في الضفادع والسمندل - اختبار فرضية مضخة الشدق الحنكي في الضفادع والسمندل، غالبًا ما يُقترح حركات مقل العيون المرتبطة بالحنك المفتوح للعب دور وظيفي في التنفس الرئوي. في "مضخة الشدق الحنكي" هذه، يتم رفع مقل العيون خلال خفض أرضية الشدق لامتصاص الهواء من خلال فتحتي الأنف، ويتم إنزال مقل العيون أثناء ارتفاع أرضية الشدق للمساعدة في ضغط الهواء داخل الرئتين. استخدمنا هنا X-Ray Reconstruction of Moving Morphology للتحقيق في حركات العين أثناء التنفس الرئوي والتغذية في الضفادع وعفريت الماء. لا تُظهر بياناتنا توافق حركات العين مع مضخة الشدق الحنكية. على العكس من ذلك، هناك ارتفاع سلبي صغير في مقل العيون عند رفع أرضية الشدق. يحدث مد داخلي للعينين فقط أثناء حركة الجسم ونقل الفرائس في الضفادع، ولكن هذا لم يلاحظ في عفريت الماء. تحتوي كل حركة عين في الضفادع على عنصر رأسي، ومتوسط جانبي، ومكون أمامي خلفي. بالنظر إلى مكون الحركة الخلفية الضعيفة بشكل مدهش في مقل العيون، فإن دورها الرئيسي في نقل الفرائس قد يكون تلصيق الفريسة عن طريق الضغط عليها ضد أرضية الشدق. وسيسهم تراجع أرضيه الشدق في الدفع الخلفي للفريسة. نظرًا لأن دراستنا لا تقدم أي دليل على وجود مضخة شدقية حنكية في الضفادع والسمندل، لا يوجد أيضًا أي دعم تجريبي لفكرة مضخة شدقية حنكية في البرمائيات Temnospondyli المنقرضة، على عكس الاقتراحات السابقة. translation by A.A. Elbassiouny ( ahmed.elbassiouny@utoronto.ca )

Movimientos oculares en ranas y salamandras: prueba de la hipótesis de la bomba bucal palatina (Eye movements in frogs and salamanders – testing the palatal buccal pump hypothesis) En ranas y salamandras, los movimientos oculares asociados con el paladar abierto a menudo se ha propuesto que desempeñan un papel funcional en la respiración pulmonar. En esta “bomba bucal palatina”, los globos oculares se elevan durante la bajada del piso bucal para inhalar por las narinas, y los globos oculares se bajan durante la elevación del piso bucal para ayudar a presionar el aire hacia los pulmones. Aquí utilizamos la Reconstrucción de Rayos X de la Morfología en Movimiento para investigar los movimientos oculares durante la respiración pulmonar y la alimentación en ranas mugidoras y ajolotes. Nuestros datos no muestran movimientos oculares que estarían de acuerdo con la bomba bucal palatina. Por el contrario, hay una pequeña elevación pasiva de los globos oculares cuando se eleva el suelo bucal. La retracción interna de los globos oculares ocurre solo durante el movimiento del cuerpo y para el transporte de presas en las ranas mugidoras, pero esto no se observó en los ajolotes. Cada movimiento ocular en las ranas mugidoras tiene un componente vertical, mediolateral y anteroposterior. Considerando el componente de movimiento posterior sorprendentemente pequeño de los globos oculares, su función principal en el transporte de presas podría ser la fijación de la presa presionándola contra el suelo bucal. La retracción del suelo bucal contribuiría entonces al empuje posterior de la presa. Debido a que nuestro estudio no proporciona evidencia de una bomba bucal palatina en ranas y salamandras, tampoco hay apoyo experimental para la idea de una bomba bucal palatina en anfibios temnospóndilos extintos, en contraste con sugerencias anteriores. translated to Spanish by Y.E. Jimenez (yordano_jimenez@brown.edu)

## Introduction

**Fig. 1:**
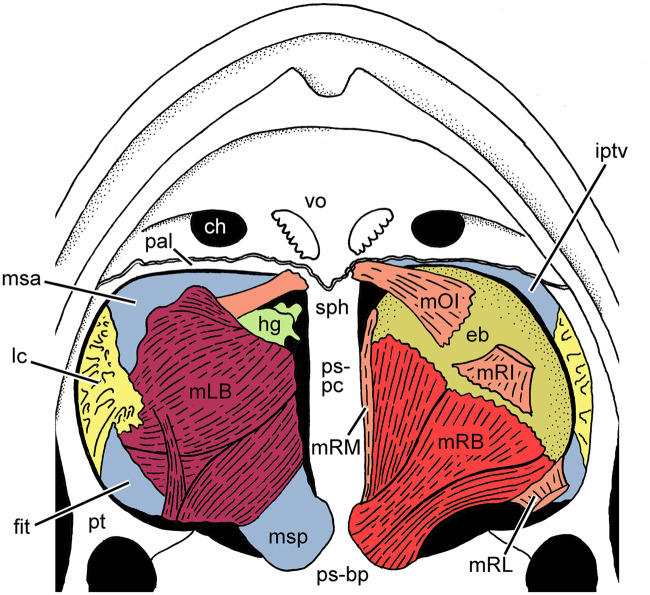
Palate of a frog (*Rana* sp.) showing the associated muscles in ventral view after removal of the buccal mucosa (redrawn and modified after Gaupp 1904). In the morphological left interpterygoid vacuity, the *m. levator bulbi* and the Harderian gland have been removed. ch, choana; eb, eyeball; fit, fascia infratemporalis; hg, Harderian gland; iptv, interpterygoid vacuity; lc, lamina cribrosa; mLB, *m. levator bulbi*; mOI, *m. obliquus inferior*; mRB, *m. retractor bulbi*; mRI, *m. rectus inferior*; mRL, *m. rectus lateralis*; mRM, *m. rectus medialis*; msa, membrana subocularis anterior; msp, membrana subocularis posterior; pal, palatine; ps-bp, basal plate of parasphenoid; ps-pc, cultriform process of parasphenoid; pt, pterygoid; sph, sphenethmoid; vo, vomer.

A diagnostic feature of lissamphibians (frogs, salamanders, and caecilians) is a palate with widely separated pterygoids (Parsons and Williams 1963). The resulting fenestrations between pterygoids and parasphenoid, the interpterygoid vacuities, are considerably enlarged especially in the “open palate” of frogs and salamanders (Duellman and Trueb 1994; Schoch 2014). This trait facilitates movements of the eyeballs into the buccal cavity (usually termed eye retraction in the literature, e.g., Schwenk 2000; Deban and Wake 2000; Levine et al. 2004). Thereby the eyes are (1) pulled in by action of the *m. retractor bulbi*, which originates on the ventral surface of the parasphenoid and is attached to the eyeball and (2) elevated by action of the *m. levator bulbi* that spans most of the interpterygoid vacuities and forms the elastic floor of the orbit (Gaupp 1904; Luther 1914; Francis 1934) (Fig. 1). Apart from frogs and salamanders, inwards drawing of the eyeballs has been described in batoids (Tomita et al. 2015) and cetaceans (Zhu et al. 2001; Bjergager et al. 2003), where it may serve mainly as protection of the eyeballs. In mudskippers, eye retraction helps the eyeballs to get remoistened (Schwab 2003; Al-Behbehani and Ebrahim 2010). Frogs and salamanders may similarly pull their eyeballs in for protection, for example, during prey capture (Nishikawa 2000) or while the animals are moving (F. Witzmann and E.L. Brainerd, personal observations). However, frogs and salamanders are unique among extant tetrapods in pulling the eyeballs down through the interpterygoid vacuities into the buccal cavity to force the prey toward the esophagus or to help to fixate it (Larsen and Guthrie 1975; Deban and Wake 2000; Schwenk 2000; Levine et al. 2004).

For a long time, a further function of the large interpterygoid vacuities and the associated eye muscles in frogs and salamanders has been hypothesized for breathing. Panizza (1845) and Gaupp (1896) proposed that movements of the eyeballs in frogs help to ventilate the lungs by buccal pumping. According to their view, the eyeballs are elevated during the lowering of the buccal floor to increase the buccal volume and to suck air in through the nares, and the eyeballs are lowered during elevation of the buccal floor to help press air from the buccal cavity into the lungs. Luther (1914) considered the role of the eyeballs for lung breathing in frogs possible, and assumed that this breathing mechanism is also present in salamanders. However, he rendered the contribution of eyeball movements to lung ventilation rather small and accessory. Francis (1934) also held the view that movements of the eyeballs assist in lung ventilation and regarded the expansion of the buccal cavity for breathing as a principal function of the *m. levator bulbi* in salamanders.

**Fig. 2:**
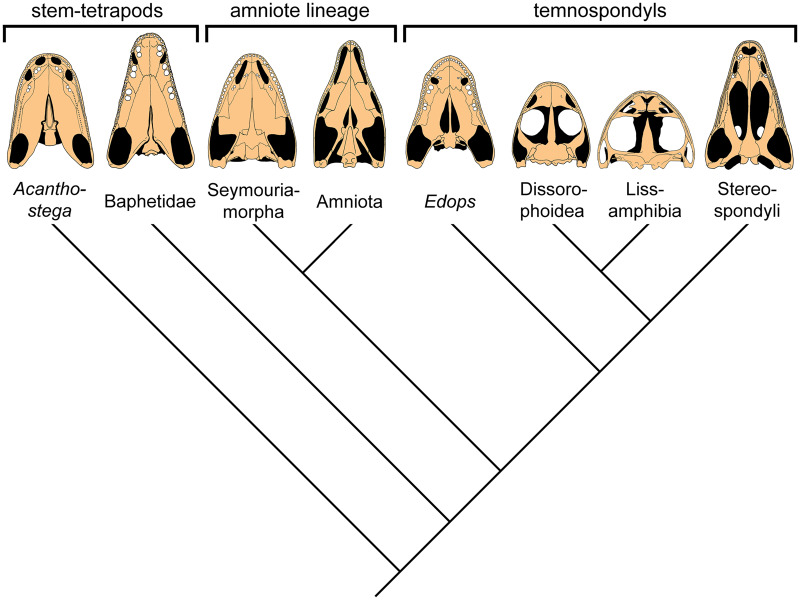
Simplified cladogram of early tetrapod relationships after Ruta and Coates (2007). Palates drawn after Romer and Witter (1942), Beaumont (1977), Reisz (1977), Duellman and Trueb (1994), Laurin (1996), Schoch and Milner (2000), Sigurdsen and Bolt (2010), and Clack (2012).

More recently, the hypothesis that the eyeballs and the interpterygoid vacuities in frogs and salamanders may contribute to lung ventilation has attracted the attention of paleontologists studying early tetrapods. Among early tetrapods, the largest and most diverse group, the temnospondyls (known from the Early Carboniferous to the Early Cretaceous), have open palates with large interpterygoid vacuities similar to extant frogs and salamanders. Lissamphibians are derived from temnospondyls (Ruta and Coates 2007; Sigurdsen and Green 2011; Schoch 2014; but see Marjanović and Laurin 2019 for a different view) and therefore, the enlarged interpterygoid vacuities of frogs and salamanders on the one hand and temnospondyls on the other hand can be regarded as a common derived character (Fig. 2) (Lautenschlager et al. 2016; Witzmann and Werneburg 2017; Witzmann and Ruta 2019). Clack (1992) was the first to propose a breathing function of the open palate in temnospondyls, while noting that the assistance of the interpterygoid vacuities in buccal pumping of extant frogs and salamanders “needs to be corroborated using modern techniques” (Clack 1992, 416–417). Laurin (2010) similarly suggested a breathing function of the palate in temnospondyls, and Clack (2012) presumed both a swallowing and a breathing function of the interpterygoid vacuities and eyeball movements in temnospondyls. Schoch (2014, 142) introduced the term “palatal buccal pump” for this presumed mode of lung ventilation in temnospondyls and lissamphibians. The presence of the palatal buccal pump in temnospondyls would indeed correspond with their proportionally large, broad heads and their rather immobile ribs, which suggest that they were buccal pumpers like lissamphibians rather than aspiration breathers (Janis and Keller 2001). Costal aspiration by contrast probably evolved in stem amniotes with a moveable ribcage and comparatively smaller heads (Janis and Keller 2001), and most of them possess a closed palate or small, slit-like interpterygoid vacuities (Fig. 2).

The present study aims to test the hypothesis of the palatal buccal pump experimentally. Prior studies have described the biomechanics of the hyobranchial buccal pump and the role of axial muscles in exhalation in lissamphibians (Brainerd et al. 1993; Brainerd and Monroy 1998; Brainerd 1998, 1999; Bennett et al. 1999; Simons et al. 2000). However, these studies did not consider the possibility of eye movements during lung ventilation. Here, we used X-Ray Reconstruction of Moving Morphology (XROMM), a set of 3D X-ray motion analysis techniques (Brainerd et al. 2010) to investigate eye movements during lung breathing in frogs and salamanders. For comparison, we recorded eye movements during prey capture and prey transport as well. Caecilians were not considered here because of their derived state of having *mm. retractor* and *levator bulbi* that are no longer connected with the eyes but instead with the tentacle (Wake 1985; Billo and Wake 1987). In caecilians, the *m. retractor bulbi* has become the retractor of the tentacle (*m. retractor tentaculi*), and the *m. levator bulbi* serves as compressor of the Harderian gland (*m. compressor tentaculi*) which belongs to the tentacle apparatus. If the hypothesized palatal buccal pump was demonstrated in frogs and salamanders, it would be reasonable to assume the presence of such a pump in temnospondyls as well because osteological correlates of frog- and salamander-like *mm. retractor* and *levator bulbi* have also been shown to be associated with the interpterygoid vacuities in temnospondyls (Witzmann and Werneburg 2017). In turn, this would support the hypothesis that temnospondyls ventilated their lungs by buccal pumping (Janis and Keller 2001).

## Materials and methods

### Animal care

Three adult American bullfrogs (*Lithobates catesbeianus*), Lc01 (male), Lc02 and Lc03 (females), with a body weight of 305, 357, and 336 g, respectively, were obtained from Charles D. Sullivan Co. Inc. in Nashville, TN, and housed at Brown University. The frogs were fed three times a week with crickets. Three adult axolotls (*Ambystoma mexicanum*), Am01, Am02, and Am03 (all males), with body weights of 87.7, 75.5, and 72.1 g, respectively, were obtained from the Ambystoma Genetic Stock Center, University of Kentucky, Lexington, KY. The axolotls were fed three times a week with dry pellets, crickets, or earthworms. All husbandry and experimental procedures were approved by the Brown University IACUC.

### Surgical implantations

**Fig. 3:**
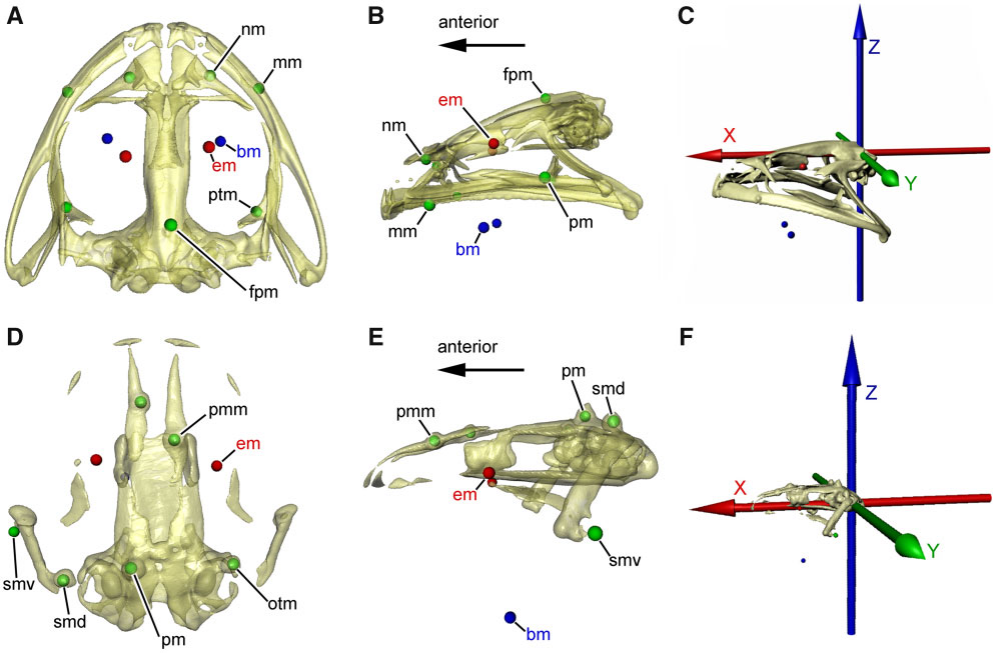
Positions of the bone and muscle markers and of the ACS in the bullfrog and the axolotl. (**A**–**C**) Bullfrog (Lc01). (A) Skull in dorsal view showing the markers in the bones (green), in the *m. levator bulbi* ventral to the eyes (red) and in the buccal floor (blue). (B) Left lateral view of same specimen as in (A). (C) Skull with attached ACS in left lateral view. (**D**–**F**) Axolotl (Am03). (D) Skull in dorsal view showing the markers in the bones (green), in the *m. levator bulbi* ventral to the eyes (red) and in the buccal floor (blue). (E) Left lateral view of same specimen as in (D). (F) Skull with attached ACS in left lateral view. bm, marker in buccal floor (*m. geniohyoideus*); em, marker in orbital floor (*m. levator bulbi*); fpm, marker in frontoparietal bone; mm, marker in maxillary bone; nm, marker in nasal bone; otm, marker in otic capsule; pm, marker in parietal bone; pmm, marker in premaxillary bone; ptm, marker in pterygoid bone; smd, marker in dorsal part of squamosal bone; smv, marker in ventral part of squamosal bone.

The bullfrogs and axolotls were surgically implanted with radio-opaque, spherical tantalum markers in bones and muscles of interest (Fig. 3). Subjects were anesthetized with MS-222 (1 g/L, neutral buffered with sodium bicarbonate). Markers were implanted into bone by drilling a hole of the same diameter as the marker, and pressing the marker into the hole. The surgical incision used to expose the bone was then closed over the markers by stitching (6/0 polyglycolic acid suture) or gluing with Vetbond tissue adhesive (3M). Markers in muscles were implanted by trocar with an inner diameter matching that of the markers, which allowed for targeted marker implantation at specific locations of interest. Surgery duration for each animal was about 2.5 h, and the animals were given an analgesic (Butorphanol, 0.2 mg kg^−1^) preoperatively and an antibiotic (Enrofloxacin, 10 mg kg^−1^) postoperatively.

#### Bullfrogs

At least five markers of 0.8 mm diameter were implanted in the cranium (left and right nasal, left and right maxilla, and posterior process of the frontoparietal). Additionally, two markers of 0.5 mm diameter were implanted in the left and right pterygoid of Lc01 and Lc03. Markers of 0.8 mm diameter were implanted in the *m. levator bulbi* ventral to the left and right eyeballs. For this, a sagittal incision of about 4 mm was made in the buccal mucosa ventral to the cultriform process of the parasphenoid, through which the hypodermic needle could be inserted to the *m. levator bulbi*. Furthermore, markers were implanted on the left and right side of the buccal floor (*m. geniohyoideus*) (Fig. 3A, B).

#### Axolotls

Due to individual variation in the extent of cranial muscle prominence, the dermal skull roofing bones were accessible to varying extents in the three axolotls. Therefore, the location of the bone markers (0.39 mm diameter) in the cranium differed slightly between the three individuals. In Am01, bone markers were implanted in the left and right premaxilla and the dorsal part of the left and right squamosal. In Am02, markers were implanted in the left and right premaxilla, the left parietal, and the ventral part of the left squamosal. In Am03, bone markers were implanted in the left and right premaxilla, the dorsal part of the left squamosal, the posterior part of the left parietal and the dorsal part of the right otic capsule (Fig. 3D, E). In all three axolotls, muscle markers of 0.5 mm diameter were implanted ventral to the left and right eyeballs (*m. levator bulbi*) and in the buccal floor anterior to the basibranchial bone (*m. geniohyoideus*).

### CT scanning and mesh models

After completion of marker implantation, computed tomography (CT) scans of each subject were taken with an Animage FIDEX veterinary CT scanner with 0.15 mm isotropic voxels. Polygonal mesh models of the cranium were reconstructed in OsiriX (v.3.2.9 64-bit, Pixmeo, Geneva, Switzerland). Marker placement was confirmed by inspecting the CT scans.

### Breathing and feeding trials

In all trials, the animals were filmed with biplanar X-ray video. Two X-ray machines (Imaging Systems and Service, Painesville, OH) were positioned for dorsoventral and lateral views of the subject as described in Brainerd et al. (2010). X-ray images were recorded with Phantom v10 high-speed cameras (Vision Research, Wayne, NJ) at 1760 × 1760 pixel resolution. The video data for this publication have been deposited in the XMAPortal (xmaportal.org) in the study “Amphibian Breathing and Feeding” with permanent ID BROWN47. Video data are stored in accordance with best practices for video data management in organismal biology (Brainerd et al. 2017).

#### Bullfrog breathing

Frog subjects were filmed during breathing while sitting in a small plastic tank (19.5 cm × 12 cm × 13.5 cm) with the floor covered with water of about 10 mm depth. X-ray settings were 100 mA for both views, with 62 kV for the lateral view and 68 kV for the dorsal view. X-ray images were recorded at 100 frames per second with a 1/1000 s shutter speed. For all three frogs, 22 sequences of breathing were recorded, in five of which the frogs also moved around.

#### Bullfrog feeding

Frog Lc03 was filmed during feeding in a plastic tank (36.8 cm × 24.8 cm × 22.2 cm) with the floor covered with water of about 15–20 mm depth. The frog was offered live crickets, and five successful feeding sequences (swallowing) were recorded from Lc03. X-ray settings were 100–125 mA for lateral view and 80–100 mA for the dorsal view, 70–75 kV for the lateral view and 70–75 kV for the dorsal view. X-ray images were recorded at 200 frames per second with a 1/500 s to 1/1000 s shutter speed.

#### Axolotl breathing

Axolotls Am02 and Am03 were filmed during breathing within a narrow tank (7 cm × 25.5 cm × 103.5 cm) in water about 10 cm deep. Lung breathing of the axolotls was induced by bubbling nitrogen into the water to evacuate oxygen. Consequently, the water became hypoxic and the axolotls came to the surface to take a gulp of air every 5–10 min. The region above the water surface was laterally shielded by a thin sheet of lead, so that the markers in the axolotls could still be traced in the X-ray videos when the snout was held above the water surface to gulp air. X-ray settings were 100 mA for both the lateral and the dorsal view, 75–90 kV for the lateral view and 75–100 kV for the dorsal view. X-ray images were recorded at 200 frames per second with a 1/500 s to 1/800 s shutter speed. For Am02 and Am03 together, 10 trials of air breathing were recorded.

#### Axolotl feeding

All three axolotls were filmed while feeding within a narrow tank (7 cm × 25.5 cm × 103.5 cm) in water about 10 cm deep. Subjects were offered crickets and live rosy minnows (*Pimephales promelas*) of 20–30 mm total length. X-ray settings were 80–90 mA for the lateral and 100–125 mA for the dorsal view, 90 kV for the lateral view and 95–105 kV for the dorsal view. X-ray images were recorded at 200 frames per second with a 1/800 s to 1/1000 s shutter speed. For all three axolotls together, 22 feeding sequences were recorded.

### Data analysis

#### XROMM

Analysis of X-ray videos including distortion correction, calibration, marker tracking, and rigid body calculations was carried out in XMALab (Knörlein et al. 2016), a software for marker-based XROMM (available at bitbucket.org/xromm/xma-lab). Standardized grid images were used for correction of fluoroscope distortion of the videos, and the 3D space was calibrated by a cube with 48 radio-opaque markers as calibration points. Mean marker tracking precision was 0.044 ± 0.035 mm (mean ± standard deviation [SD], *n* = 165 pairwise distances of markers within rigid bodies) in the bullfrogs with the lowest SD being 0.023 mm and highest 0.148 mm, and 0.048 ± 0.021 mm (*n* = 37 pairwise distances) in the axolotls with lowest being 0.035 mm and highest 0.132 mm. The cranium was treated as a single rigid body object for all subjects. Rigid body transformations and translations of single markers (in muscle ventral to the eyeballs and in the buccal floor) were applied to the polygonal mesh bone models in Autodesk Maya (2014, Autodesk Inc., San Rafael, CA) using XROMM MayaTools (available at bitbucket.org/xromm/xromm_mayatools) to animate movements of the models during breathing and feeding.

#### Anatomical coordinate systems

A single anatomical coordinate system (ACS) of the cranium with *x*-, *y*- and *z*-axes defined as orthogonal to each other was created in Autodesk Maya for each bullfrog and axolotl. The color convention is red for the *x*-axis, green for the *y*-axis, and blue for the *z*-axis (Fig. 3C, F). The ACS was placed at the posterior edge of the cranium, which was used as the reference bone, between the paired exoccipitals (Fig. 3C, F). The *x*-axis was oriented anteroposteriorly (pointing in the anterior direction) along the long axis of the cranium in the midsagittal plane and passing through the foramen magnum and dorsal to the parasphenoid, the *y*-axis was oriented mediolaterally (pointing to the left) parallel to the transverse axis of the skull, and the *z*-axis was oriented dorsoventrally (pointing in the dorsal direction). We took measurements of the 3D translations of the two markers ventral to the eyeballs (henceforth called eye markers) and of the two markers in the buccal floor (buccal floor markers) along each of the three ACS axes in Autodesk Maya. If the palatal buccal pump was present in bullfrogs and axolotls, the eye markers should exhibit during lung breathing (1) positive *z*-axis translation (i.e., elevation of the eyes) in correlation with negative *z*-axis translation of the buccal floor markers (i.e., depression of the buccal floor), and (2) negative *z*-axis translation (i.e., depression of the eyes) in correlation with positive *z*-axis translation of the buccal floor markers (i.e., elevation of the buccal floor).

## Results

### Buccal floor and eye motions during breathing and body movement in the bullfrog

#### Breathing

**Fig. 4:**
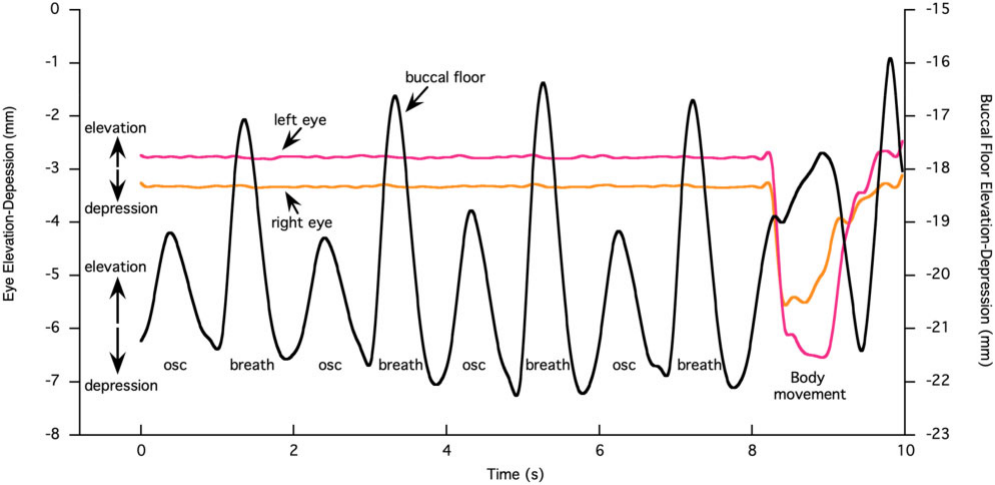
Elevation and depression of the eyes and buccal floor during buccal oscillation, lung ventilation, and body movement in a bullfrog. Upward deflection of the motion traces is elevation and downward deflection is depression. The buccal floor elevates and both eyes depress during body movement. osc, buccal oscillation; breath, buccal pump for lung ventilation.

Figure 4 shows the movements along the *z*-axis of the left and right eyes and the buccal floor (one marker selected) of a bullfrog while it is sitting still and breathing for ∼8 s, before it is moving its body at the end of the sequence. While the frog is sitting still, the movement of the buccal floor shows eight peaks and valleys, with alternating larger and smaller peaks. Each peak represents elevation of the buccal floor, and each valley represents its depression. The small peaks illustrate buccal oscillation (*sensu*Brainerd and Owerkowicz 2006) during which air is drawn into the buccal cavity through the nostrils (buccal floor depression) and is pumped out again (buccal floor elevation) through the nostrils without entering the lungs. By contrast, each large peak illustrates breathing during which the pronounced upward movement of the buccal floor presses air into the lungs. This can also be observed by inflation of the lungs in the X-ray videos. The amplitude of the dorsoventral buccal floor movement during breathing is on average more than twice the amplitude of the buccal floor movement during oscillation (5.5 mm vs. 2.3 mm, with SD of 1.850 and 0.448, respectively, calculated based on 65 measurements of right and left buccal floor movement in all three individuals, see Supplementary Table ST1). During this sequence of breathing and buccal oscillation, both the left and right eyes do not move along the *z*-axis. There is only a minimal elevation of the eyeballs at the same time when the buccal floor is elevated during breathing (Fig. 4). The mean eye elevation during buccal compression for each individual and left and right eyeballs together is 0.062 ± 0.027 mm based on 52 breaths (see Supplementary Table ST2).

#### Body movement

**Fig. 5:**
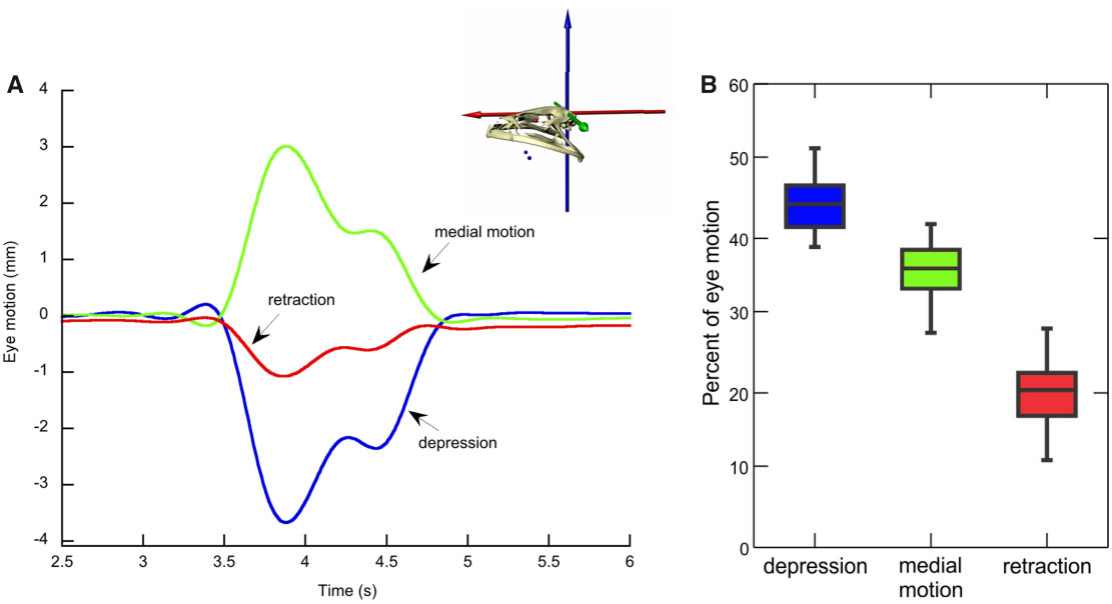
Eye translations in 3D during body motion in bullfrogs. (**A**) Example trace with inset of the ACS. Eye depression in blue, medial motion in green, and retraction in red. (**B**) Percentage of the total motion from each translational degree of freedom.

During body movement at the end of the sequence shown in Fig. 4, both eyes are depressed, whereby the left eye moves 3.75 mm and the right eye 2.3 mm along the *z*-axis. Simultaneously, the buccal floor is elevated by a magnitude that is intermediate of buccal oscillation and breathing and is kept elevated during the time the eyeballs are depressed. Figure 5A shows the translation of the right eyeball along the *x*-, *y*-, and *z*-axes during body movement in another sequence. The ventral movement of the eyeball (negative *z*-translation) constitutes the main component of the 3D motion, followed by the medial movement (positive *y*-translation because the *y*-axis is directed to the left), whereas the posterior component (negative *x*-translation or eye retraction *sensu stricto*) constitutes the smallest proportion of total movement. The percentage from each of the three translations (along *x*-, *y*-, and *z*-axes) with respect to the total motion (calculated as the sum of the three translations) for both eyes and all individuals (based on 16 eye inward movements, see Supplementary Table ST3) is 44.5 ± 3.61% for *z*-translation, 35.8 ± 4.29% for *y*-translation, and 19.7 ± 4.19% for *x*-translation (Fig. 5B).

### Buccal floor and eye motions during prey transport in the bullfrog

**Fig. 6:**
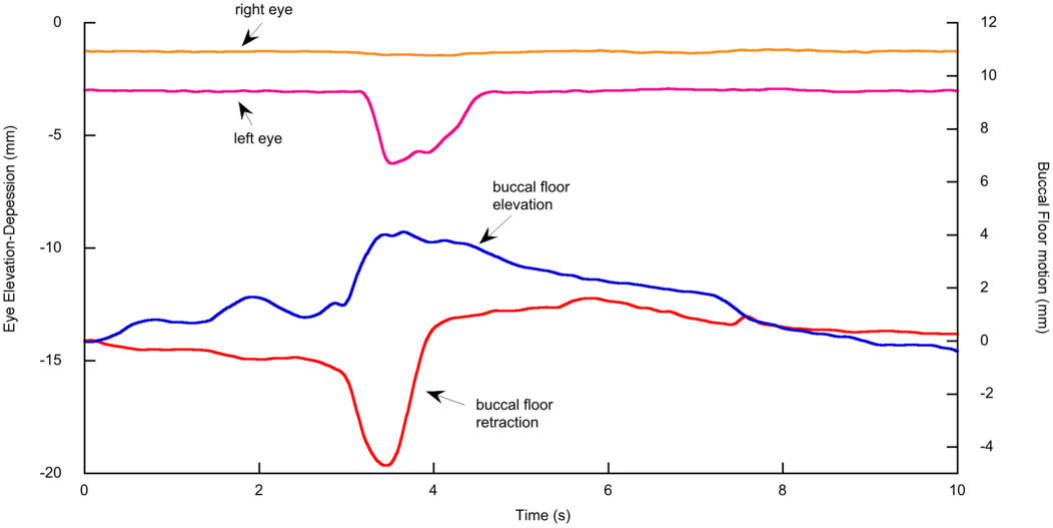
Depression of the left eye and elevation and retraction of the buccal floor during prey (cricket) transport in a bullfrog. Upward deflection of the motion traces is elevation or protraction and downward deflection is depression or retraction.

For comparison with the pattern recorded during body movement, motions of eyes and buccal floor were also studied during prey transport in the bullfrog. Figure 6 shows *z*-translation (dorsoventral movements) of the eyeballs and *z*- and *x*-translations (dorsoventral and anteroposterior movements) of the buccal floor during transport of a cricket. In this trial, only the left eyeball was pulled in, whereas the right one did not move. Concomitantly with eye depression, there is peak elevation and retraction of the buccal floor. In contrast to eye depression, which takes place abruptly, the buccal floor is already slightly elevated and retracted prior to the peak, and the buccal floor is depressed slowly after peak elevation.

### Buccal floor and eye motions during lung breathing and feeding in the axolotl

#### Lung breathing

**Fig. 7:**
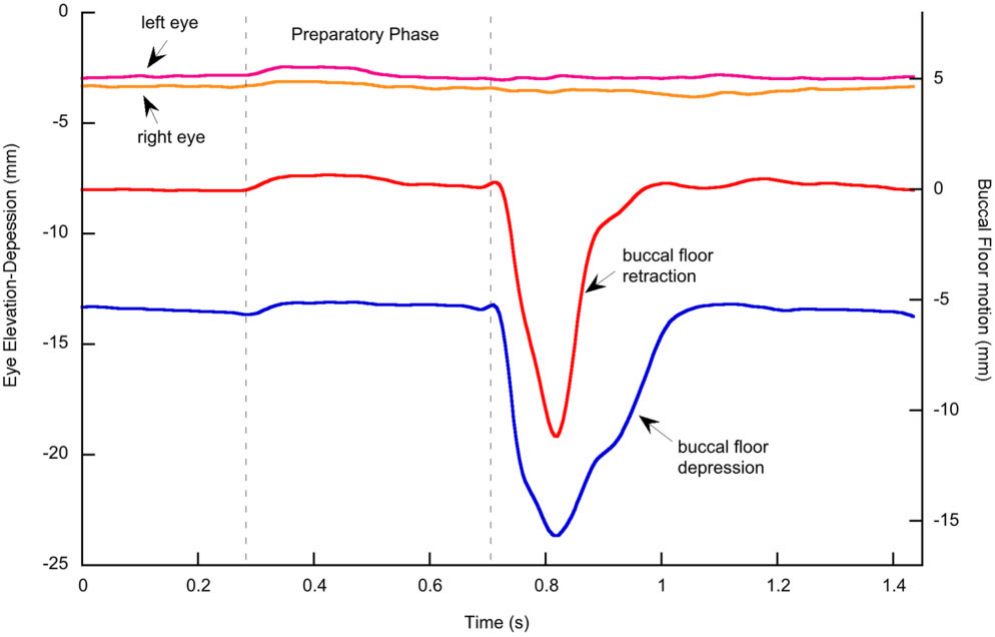
Eye and buccal floor motions during an air gulp in an axolotl. A slight preparatory phase of buccal floor elevation and protraction produces slight elevation of the eyes. The eyes do not move in any consistent manner during the air gulp.

Figure 7 shows motions of the left and right eyeballs along the *z*-axis (dorsoventral motion) and of the buccal floor along the *z*- and *x*-axes (dorsoventral and anteroposterior motion) during lung breathing in an axolotl. When the axolotl approaches the water surface, the buccal floor is elevated and protracted, and it is kept in this position (preparatory phase). Then the snout breaks through the water surface and the buccal floor is rapidly depressed and retracted, and air is sucked in through the open mouth. The buccal floor is quickly elevated and protracted again, and the air is pressed into the lungs. Concomitantly with buccal floor elevation, the eyeballs are slightly elevated during the preparatory phase. During retraction and depression of the buccal floor, the eyeballs display minute dorsoventral movements, but not in a consistent manner and show no correlation with the movements of the buccal floor.

#### Feeding

**Fig. 8:**
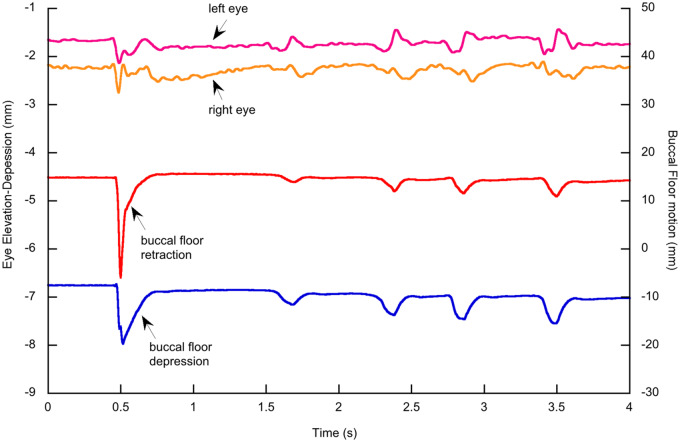
Eye and buccal floor motions during suction feeding and prey (cricket) processing in an axolotl. The eyes depress slightly (<1 mm) during the suction strike, and then move slightly and inconsistently during prey processing. Note the different scales for eye (left axis) and buccal motions (right axis).

For comparison with breathing, we also recorded buccal floor and eye motion during feeding of the axolotls. Figure 8 shows translations of the left and right eyeballs along the *z*-axis (dorsoventral movements) and translations of the buccal floor along the *z*- and *x*-axes (dorsoventral and anteroposterior motion) during capture and processing of a cricket. The suction strike includes a rapid depression and retraction of the buccal floor, and buccal floor retraction is larger than buccal floor depression. The eyes depress slightly during the suction strike and their maximum depression (<1 mm) coincides with maximum depression and retraction of the buccal floor. The figure illustrates four smaller depressions and retractions of the buccal floor during the subsequent food processing. During this phase, the eyes move slightly and inconsistently. During the first two movements of the buccal floor, both eyes are elevated to some degree, but while the left eye is elevated also in the third and fourth buccal floor movements, the right eye is depressed.

## Discussion

### Eye movements during breathing

The main aim of this work was to test the hypothesis of the palatal buccal pump in frogs and salamanders, that is, the contribution of eyeball movements to buccal pumping. If the palatal buccal pump had contributed to buccal pumping, we would expect that the eyes to be elevated during depression of the buccal floor, and depressed during elevation of the buccal floor. Our data collected during air breathing by buccal pumping do not support the presence of a palatal buccal pump in bullfrogs and axolotls. Not only are there no eye movements that would be in accordance with this pump, there is also a small elevation of the eyeballs in bullfrogs when the buccal floor is raised, and this is opposite to the direction of eye movement as hypothesized for the palatal buccal pump. Considering the tiny magnitude of eye elevation that occurs simultaneously with the elevation of the buccal floor, these eye movements can be considered passive and driven by the buccal floor movements rather than by eye muscles. The movements of the eyes during lung breathing of the axolotls are likewise minimal (<1 mm) and show no consistent pattern. There is no elevation of the eyeballs during buccal floor retraction and depression as the palatal buccal pump hypothesis would predict. Similar to the bullfrogs, the slight upward movement of the eyes in axolotls during buccal elevation in the preparatory phase of lung breathing suggests that eye movements are largely passive and driven by pressure changes caused by movements of the buccal floor. Furthermore, if the eye movements had the function to assist in enlargement and reduction of buccal volume to suck in air and to press it into the lungs, the magnitude of the movements should be distinctly larger (and in the opposite direction).

### Eye and buccal floor movements during body motion

Whereas drawing the eyes inwards during body motion was not observed in axolotls, bullfrogs frequently pull their eyes in as they commence body movement. This might be connected with protection of their protruding eyeballs, similar to the depression of the eyes during the feeding strike (Nishikawa 2000; F. Witzmann and E.L. Brainerd, personal observations). The inward drawing of the left and right eyes is often not symmetrical (see e.g., Fig. 4). These discrepancies of left and right eye movements may be increased by the fact that the left and right eye markers are not exactly symmetrically placed below the eyeballs. Our analysis of eyeball motions along the three axes of the ACS shows that each eye movement has a vertical component (translation along the *z*-axis), a mediolateral component (translation along the *y*-axis), and an anteroposterior component (translation along the *x*-axis). Surprisingly, as frogs pull their eyeballs in, the medial movement is nearly as large as the ventral one, whereas the posterior movement is distinctly smaller (Fig. 5B). Strictly speaking, the common term “eye retraction” is thus not correct, because the posterior component (retraction *sensu stricto*) is so small. Interestingly, bullfrogs elevate the buccal floor for the time the eyeballs are drawn in during body movements.

### Eye and buccal floor motions during feeding

Our data (albeit limited) on bullfrog feeding (*n *=* *1 individual) show that the buccal floor is elevated while the eyes are pulled in during feeding similar to the cases when the body moves (see above). Considering the rather weak posterior motion component of the eyeball, their main role in prey transport might be fixing the prey by pressing it against the buccal floor rather than forcing the prey backwards to the esophagus. The retraction of the buccal floor (Fig. 6) would then contribute to the posterior push of the prey in the buccal cavity. Our observations of asymmetrical eye movements during prey transport in bullfrogs are consistent with Levine et al. (2004) who reported both unilateral and bilateral movements of the eyeballs during swallowing in the leopard frog. Unilateral movements of the eyes may depend on the location of prey in the buccal cavity, that is, it is located on one side of the mouth (Levine et al. 2004). However, this was not observed to be accompanied by unilateral movements of the buccal floor.

Whereas our data and the work of Levine et al. (2004) clearly suggest a function of the eyeballs during prey transport in frogs, this cannot be demonstrated in the axolotl. The depression of the eyeballs during the suction strike (Fig. 7) is minute and occurs exactly when the buccal floor is depressed and retracted, suggesting that these movements are passive and driven by the low pressure in the buccal cavity during buccal expansion. Also, in prey transport the eyes seem to be pushed around passively rather than showing a consistent movement pattern, and the amplitude of their movement is tiny (around 0.5 mm). Therefore, the eyeballs appear to play no functional role in axolotl feeding. However, this might be due to the fact that the axolotl is a perennibranchiate salamander with a larval morphology, and the eyeballs might indeed contribute to swallowing performance in metamorphosed salamanders. Based on external observations, Larsen and Guthrie (1975) reported eye depression probably correlated with prey transport in adult specimens of the closely related tiger salamander (*A.tigrinum*) in which the eyeballs enlarged during metamorphosis. This could be tested experimentally in future research with 3D X-ray motion analysis.

### Implications for extinct temnospondyls

Non-lissamphibian temnospondyls not only have open palates with enlarged interpterygoid vacuities, similar to frogs and salamanders, their interpterygoid vacuities also seem to be associated with frog- and salamander-like *mm. retractor* and *levator bulbi* muscles based on osteological correlates on the palatal bones and the neurocranium (Witzmann and Werneburg 2017). Consequently, temnospondyls were maybe able to move their eyeballs posteroventrally and medially into the buccal cavity for protection of their eyes and for assistance in swallowing. However, because our study provides no evidence for a palatal buccal pump in frogs and salamanders, and because such a kind of pump has never been reported in any other living tetrapod or fish, we find no experimental support for the idea of a palatal buccal pump in temnospondyls as has been suggested previously (Clack 1992, 2012; Laurin 2010; Schoch 2014). In spite of this, it is most likely that temnospondyls were buccal pumpers (Janis and Keller 2001) but they changed the volume of the buccal cavity solely by movements of the buccal floor.

## Data availability

Data are available from XMAPortal (xmaportal.org) in the study “Amphibian Breathing and Feeding” with permanent ID BROWN47.

## Supplementary Material

Supplementary_Material_obz011
